# Large Optical Gain AlInN-Delta-GaN Quantum Well for Deep Ultraviolet Emitters

**DOI:** 10.1038/srep22983

**Published:** 2016-03-10

**Authors:** Chee-Keong Tan, Wei Sun, Damir Borovac, Nelson Tansu

**Affiliations:** 1 Center for Photonics and Nanoelectronics, Department of Electrical and Computer Engineering, Lehigh University, Bethlehem, PA 18015 USA

## Abstract

The optical gain and spontaneous emission characteristics of low In-content AlInN-delta-GaN quantum wells (QWs) are analyzed for deep ultraviolet (UV) light emitting diodes (LEDs) and lasers. Our analysis shows a large increase in the dominant transverse electric (TE) polarized spontaneous emission rate and optical gain. The remarkable enhancements in TE-polarized optical gain and spontaneous emission characteristics are attributed to the dominant conduction (C)-heavy hole (HH) transitions achieved by the AlInN-delta-GaN QW structure, which could lead to its potential application as the active region material for high performance deep UV emitters. In addition, our findings show that further optimizations of the delta-GaN layer in the active region are required to realize the high performance AlInN-based LEDs and lasers with the desired emission wavelength. This work illuminates the high potential of the low In-content AlInN-delta-GaN QW structure to achieve large dominant TE-polarized spontaneous emission rates and optical gains for high performance AlN-based UV devices.

III-Nitride semiconductor alloys have been widely implemented for solid state lighting applications over the past decade^1^,^2^,^3^,^4^,^5^. The progresses in material epitaxy and device innovations of GaN-based semiconductor alloys have led to a revolution in lighting technology^6^, in which the advances in the GaN-based light emitting diodes (LEDs) were recently recognized by the Nobel Prize in Physics in 2014^7^. In recent years, III-Nitride deep ultraviolet (UV) LEDs and laser diodes have attracted much interests due to the prospective applications in sterilization, water purification and waste water treatment^8^,^9^,^10^,^11^,^12^,^13^,^14^,^15^,^16^,^17^,^18^,^19^,^20^,^21^,^22^,^23^,^24^,^25^,^26^,^27^,^28^,^29^,^30^,^31^. Extensive studies have been focused in the use of AlGaN-based quantum well (QW) active region for the UV emitters, attributed to the direct band gap property of the AlGaN alloy and the band gap coverage in the UV spectral regime from 210 nm up to 370 nm.

In comparison to the advances in the InGaN-based LEDs in the blue and green emitting regime^32^,^33^,^34^,^35^,^36^,^37^,^38^,^39^,^40^,^41^,^42^,^43^,^44^, the AlGaN-based QW DUV-LEDs still suffer from the low external quantum efficiency (EQE) issue^8^,^9^,^10^,^11^. The low EQE in the AlGaN LEDs is attributed to several factors including the low material growth quality due to high threading dislocation density^12^, poor hole concentration^13^, poor light extraction efficiency^14^, fundamental valence bands crossover issue^15^,^16^,^17^ and the charge separation issue due to the polarization effect^5^,^18^. These fundamental issues have resulted in the low EQE of ~10%, despite the tremendous efforts devoted to enhancing the efficiency of the AlGaN DUV LED^11^. Most of the issues encountered in the AlGaN DUV LED are nonetheless similar to those of InGaN LEDs, except the fundamental valence bands crossover issue present in the AlGaN active region material.

In the low Al-content AlGaN QW, the heavy hole (HH)/light hole (LH) band edge is located above the crystal field split-off hole (CH) band edge. The increase of the Al-content in the AlGaN QW will result in the reduction of band-edge energy separation between the CH band and HH/LH band, eventually leading to the crossover between CH band and HH/LH band. Thus, for high Al-content AlGaN QW that is needed for deep UV emission, the CH band edge is located above the HH/LH band. Recent works on valence bands crossover issue showed that the use of high Al-content AlGaN quantum well (QW) for deep UV emitter led to large transverse-magnetic (TM)-polarized gain but small transverse-electric (TE)-polarized gain. This phenomenon is primarily driven by the dominant conduction (C) – crystal field split-off hole (CH) transition in the AlGaN QW, due to the arrangement of the CH band as the uppermost valence band^16^. Note that the TE-polarized emission refers to the light propagating perpendicular to the QW plane, while the the TM-polarized emission refers to the in-plane light propagating parallel to the QW plane. However, the TM-polarized emission of the AlGaN QW is undesirable for the deep UV top emitting devices due to the difficulty of extracting the TM-polarized light from the top surface of the device, resulting in low light extraction efficiency^19^. The inefficient light extraction in combination with the weak TE polarized emission from valence band crossover issue therefore leads to low quantum efficiency in the high Al-content AlGaN QWs. Various studies targeting the polarization switching in the AlGaN QW for dominant TE-polarized emission have been carried out by investigating the effect of variables such as Al composition of the QW, the QW thickness and the strain in the QW^19^,^22^. A different approach has also been suggested by employing AlGaN-delta-GaN QW structure, in which the valence bands are rearranged, leading to dominant TE-polarized emission^23^,^24^. These approaches nonetheless focused in the use of AlGaN alloy in the active region, while the approach on using different III-Nitride UV materials is relatively unexplored. Identifying and developing the potential of alternative UV materials will be critical towards the progress in deep UV emitters.

Recently, there has been an emerging interest in using the AlInN alloy as the active region for deep UV laser diodes (λ ~ 250 nm)^28^, attributed to the full band gap coverage of the material in the UV spectrum. However, the literature on AlInN QW as the active region for deep UV emitters is still highly limited up to present^28^,^29^, despite that the AlInN alloy has been extensively studied for employing in the visible light emitting^37^,^38^,^44^ and power electronics applications^45^,^46^. Our preliminary findings indicate that the use of conventional AlInN QW results in small dominant TE-polarized gain and spontaneous emission rates, albeit the polarization switching occurring at high Al-content (~ 90%) in the QW^29^. Thus, the innovations in the active region design and the understanding in the physics of optical properties of the AlInN QW will be important for enhancing the corresponding spontaneous emission and gain characteristics for deep UV emission.

In this work, numerical analyses were carried out to investigate the spontaneous emission properties and the optical gain of the AlInN-delta-GaN quantum well (QW) with AlN barriers for the deep UV emitters. The band diagrams and carrier wave functions were calculated based on a 6-band ***k·p*** formalism in which the valence band mixing, strain, polarization fields and carrier screening effects were taken into account^39^, with the band parameters obtained from refs 39 and 47. The spontaneous rate and gain are then calculated taking into account transitions between all populated conduction and valence subbands. Our study shows tremendous increment of the dominant TE-polarized spontaneous emission rate and the optical gain of AlInN-delta-GaN QW with low In-content as compared to the conventional AlInN quantum well with AlN barriers. In addition, the effect of the delta-GaN layer thickness is investigated, which revealed the importance of optimizing the delta-GaN layer in the AlInN-delta-GaN QW in order to obtain high optical gain for the desired emission wavelength.

## Concept

**Figure 1:**
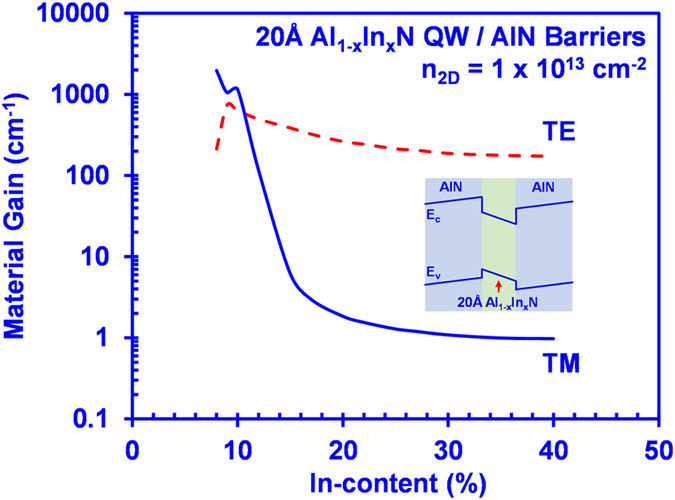
TM peak material gains (g^TM^_peak_) and TE peak material gains (g^TE^_peak_) as a function of In-content (x) for 20 Å AlInN conventional QW with AlN barriers for *n*_*2D*_ = 1 × 10^13^ cm^−2^.

Figure 1 shows the material gains calculated for a 2 nm conventional Al_1–x_In_x_N QW with AlN barriers with In-content (x) ranging from 8% to 40% at a sheet carrier density (*n*_*2D*_) of 1 × 10^13^ cm^−2^. As shown in fig. 1, large TM-polarized material gains (g^TM^_peak_) can be achieved for the AlInN QW with low In-content (~8–10%) in the ~220 nm spectral regime, while the corresponding TE-polarized material gains are relatively low. Both the TE- and TM-polarized gain show a reducing trend for increasing In-content beyond 10%, which is caused by the lower momentum matrix element at higher In-content of Al_1–x_In_x_N QWs. Even though the g^TE^_peak_ is dominant over the g^TM^_peak_ as shown in fig. 1, both the TE- and TM-polarized g_peak_ for high In-content AlInN QW are relatively low (<500 cm^−1^). The optical gain of AlInN QW can be improved by enhancing the electron-hole wavefunction overlap through the reduction of the QW thickness. However, higher In-content required in the AlInN QW to compensate the wavelength blueshift will potentially lead to lower material quality, resulting in lower performance for the UV emitters. Thus, the pursuit of the AlInN-delta-GaN QW would be important towards obtaining large optical gain and high spontaneous emission rates in the UV emitters.

**Figure 2:**
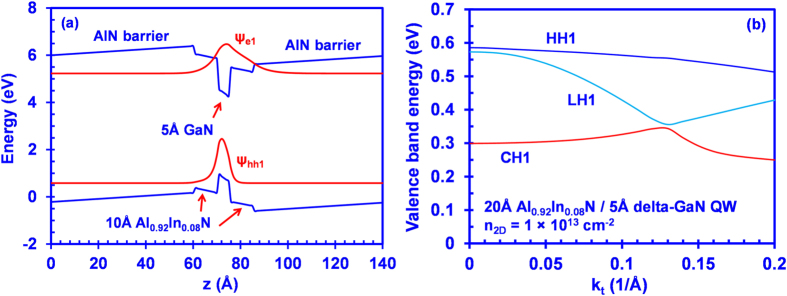
(**a**) Energy band lineup of AlInN-delta-GaN QW along with the carrier wavefunctions for both the electron and heavy hole in the conduction and valence band, respectively, with the calculation performed self-consistently at sheet carrier density (*n*_*2D*_) = 1 × 10^13^ cm^−2^. (**b**) Valence band structure of the 20 Å Al_0.92_In_0.08_N/5 Å delta-GaN QW with AlN barriers.

Figure 2(a) presents the energy band lineup of the 20 Å Al_0.92_In_0.08_N/5 Å delta-GaN QW with AlN barriers along with the carrier wavefunctions of the first conduction and valence sub-bands at sheet carrier density *n*_*2D*_ of 1 × 10^13^ cm^−2^. The band lineups for all the AlInN-based QW structures are calculated self-consistently taking into account the carrier screening effect. Note that the gain and spontaneous emission rates were also calculated self-consistently for all the conventional AlInN QW and AlInN-delta-GaN QW structures at different carrier densities, which is similar to the treatment shown in previous work^39^. The charge neutrality requirement is applied to the structure including the well and barriers. As shown in fig. 2(a), the insertion of a delta-GaN layer traps the electron and hole wavefunctions in the center of the active region, resulting in large electron-hole wavefunction overlap. The change of In-content for the AlInN layers will result in the change of electron-hole wavefunction overlap accordingly. As an example, the electron-hole wavefunction overlap in Al_0.83_In_0.17_N-delta-GaN QW exhibits ~69%, while the electron-hole wavefunction overlap in Al_0.92_In_0.08_N-delta-GaN QW exhibits ~82%. Note that in the conventional AlInN QW, due to the built-in polarization field the electron and hole wavefunctions being spatially separated, a lower electron-hole wavefunction overlap is observed.

Figure 2(b) provides the valence band structure of 20 Å Al_0.88_In_0.12_N/5 Å delta-GaN QW with AlN barriers. By employing the AlGaN-delta-GaN QW, the strong valence band mixing effect introduced by the delta-GaN layer results in valence band rearrangement, leading to higher heavy hole (HH1) band and light hole (LH1) band in comparison to that of the crystal-field split-off hole (CH1) band. The large energy separation of 0.27 eV at the gamma-point between the HH1 and CH1 subbands thus results in dominant TE-polarized emission in the AlInN-delta-GaN QW structure.

## Results and Discussion

**Figure 3:**
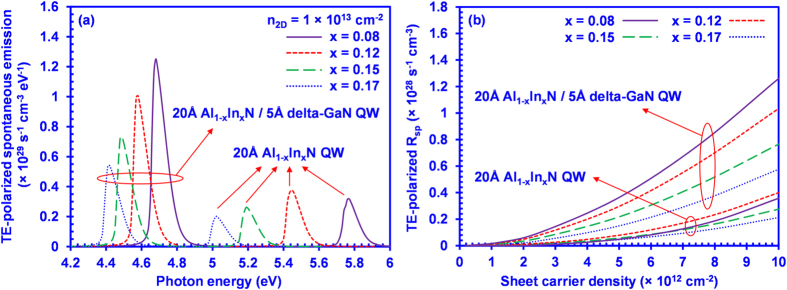
(**a**) TE-polarized spontaneous emission spectra as a function of photon energy at sheet carrier density of 1 × 10^13^ cm^−2^ at T = 300 K and (**b**) TE-polarized spontaneous emission rate per unit volume as a function of sheet carrier density from 2.5 × 10^11^ cm^−2^ to 1 × 10^13^ cm^−2^, for 20 Å Al_1–x_In_x_N/5 Å delta-GaN QW and 20 Å Al_1–x_In_x_N QW.

Figure 3(a) illustrates the TE-polarized spontaneous emission spectra as a function of photon energy for the 20 Å Al_1–x_In_x_N/5 Å delta-GaN QWs and the conventional Al_1–x_In_x_N QWs at a sheet carrier density (*n*_*2D*_) of 1 × 10^13^ cm^−2^ at T = 300 K. The peak TE-polarized spontaneous emission rates of the 20 Å Al_1–x_In_x_N/5 Å delta-GaN QW range from 1.25 × 10^29^ s^−1^ cm^−3^ eV^−1^ for 20 Å Al_0.92_In_0.08_N/5 Å delta-GaN QW to 0.54 × 10^29^ s^−1^ cm^−3^ eV^−1^ for 20 Å Al_0.83_In_0.17_N/5 Å delta-GaN QW, which are nearly 4 times larger than those of 20 Å Al_1–x_In_x_N conventional QWs. Our analysis indicates that the dominant TE-polarized spontaneous emission rate is significantly enhanced by applying the delta-GaN layer into the Al_1–x_In_x_N QW structure. In comparison to the redshift from 215 nm to 247 nm in 20 Å conventional Al_1–x_In_x_N QWs, the spontaneous emission spectra show the relatively smaller redshift of emission wavelength from 265 nm to 280 nm when the In-content increases from 8% to 17% in the 20 Å Al_1–x_In_x_N/5 Å delta-GaN QWs. Note that the peak spontaneous emission rate increases with respect to the reduction of In-content in AlInN-delta-GaN QW. This phenomenon is attributed to the increase of electron-hole wavefunction overlap in the active region, as a result of the decreased compressive strain when the In-composition of AlInN layer reduces. Our study shows the potential of applying AlInN-delta-GaN QW with low In-content to achieve sufficient spontaneous emission rates for high performance UV LEDs.

Figure 3(b) shows the comparison of the spontaneous emission rate per unit volume (*R*_*sp*_) with respect to the sheet carrier density between the 20 Å Al_1–x_In_x_N/5 Å delta-GaN QW and the conventional 20 Å Al_1–x_In_x_N QW. As shown in fig. 3(b), the R_sp_ for the 20 Å Al_1–x_In_x_N /5 Å delta-GaN QW is significantly larger than that of the conventional Al_1–x_In_x_N QW, as sheet carrier density (*n*_*2D*_) increases from 2.5 × 10^11^ cm^−2^ to 1 × 10^13^ cm^−2^. The larger *R*_*sp*_ shown in the 20 Å Al_1–x_In_x_N/5 Å delta-GaN QW is attributed to the large and dominant TE-polarized spontaneous emission in the active region.

**Figure 4:**
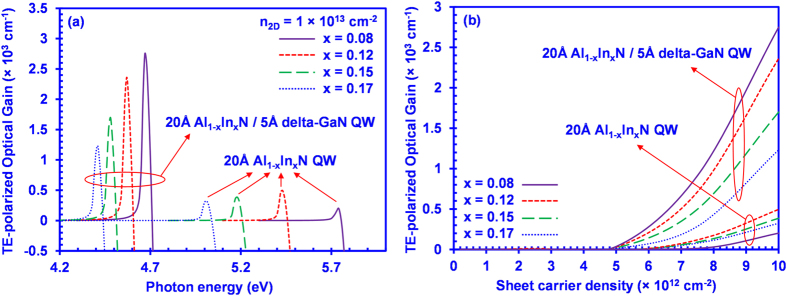
(**a**) TE-polarized optical gain spectra at *n*_*2D*_ = 1 × 10^13^ cm^−2^, and (**b**) TE-polarized peak optical gain as a function of sheet carrier density for 20 Å Al_1–x_In_x_N/5 Å delta-GaN QW and for 20 Å Al_1–x_In_x_N QW.

Figure 4(a) shows the TE-polarized optical gain spectra for the 20 Å Al_1–x_In_x_N/5 Å delta-GaN QWs as well as the conventional 20 Å Al_1–x_In_x_N QWs at an *n* of 1 × 10^13^ cm^−2^. As shown in fig. 4(a), the peak TE-polarized optical gain of the 20 Å Al_1–x_In_x_N/5 Å delta-GaN QW yields an increasing trend with the reduction of In-composition (x). The peak TE-polarized optical gain reaches ~ 2750 cm^−1^ for the 20 Å Al_1–x_In_x_N/5 Å delta-GaN QW with 8% In-content, whereas the 20 Å Al_1–x_In_x_N/5 Å delta-GaN QWs with x = 12%, 15% and 17% have a lower TE optical gain corresponding to around 2400 cm^−1^, 1700 cm^−1^ and 1230 cm^−1^, respectively. In comparison to the conventional 20 Å Al_1–x_In_x_N QWs, the 20 Å Al_1–x_In_x_N/5 Å delta-GaN QW yields around 6 times the increase in its peak TE-polarized optical gain. The enhancement of the TE-polarized optical gain can be attributed to a significant increase in the optical transition matrix element in the 20 Å Al_1–x_In_x_N/5 Å delta-GaN QW structure. Similar to the phenomena shown in the spontaneous emission spectra for the AlInN-delta-GaN QW, the larger enhancement of the optical gain as In-content reduces in the AlInN-delta-GaN QWs is attributed to the larger optical matrix element. Note that in contrast to the the AlInN-delta-GaN QW, the TE-polarized optical gain is smaller in the conventional AlInN QW with In-content of 8% in comparison to that of AlInN QW with In-content of 12%. This is attributed to the fact that the conventional AlInN QWs with In-content of 8% suffers from the valence band crossover issue, which leads to a small value of TE-polarized optical gain.

In fig. 4(b), the peak TE-polarized optical gain of the 20 Å Al_1–x_In_x_N/5 Å delta-GaN QW and the conventional 20 Å Al_1–x_In_x_N QWs is plotted as a function of sheet carrier density ranging from *n*_*2D*_ = 2.5 × 10^11^ cm^−2^ to *n*_*2D*_ = 1 × 10^13^ cm^−2^. The figure shows that the TE-polarized optical gain of the 20 Å Al_1–x_In_x_N/5 Å delta-GaN QWs structure has a significant increase over that of the conventional 20 Å Al_1–x_In_x_N QWs at various sheet carrier densities ranging from 2.5 × 10^11^ cm^−2^ to 1 × 10^13^ cm^−2^. Our analysis demonstrates the importance of applying the delta-GaN layer in the conventional AlInN QW, in order to enhance the dominant TE-polarized optical gain for high performance deep UV lasers.

The potential of the AlInN-delta-GaN QW as the active region for laser diode applications can be assessed through the investigation of the threshold properties of the QW. In our analysis, an optical confinement factor (Γ_opt_) of 0.02^24^,^31^ is used for the AlInN-delta-GaN QW lasers. The internal loss is assumed to be 14 cm^−1^ for the AlInN-delta-GaN QW laser, while the laser cavity length is assumed as 500 μm with a mirror loss of 11 cm^−1^^24^. The resulting threshold gain (g_th_) for the AlInN-delta-GaN laser is ~1250 cm^−1^, with corresponding modal gain of 25 cm^−1^. As an example, as shown in fig. 4(b), the threshold sheet carrier density (*n*_*2D_th*_) is 8.4 × 10^12^ cm^−2^ for Al_0.88_In_0.12_N-delta-GaN QWs. On the other hand, the TE-polarized gain obtained at similar carrier density in conventional AlInN QW is only ~220 cm^−1^. This indicates that significantly higher carrier density will be required for the conventional QW to achieve g_th_ of ~1250 cm^−1^. The reduction in threshold carrier density via the use of delta-based QW is essential for suppressing the non-radiative recombination current, namely monomolecular (~ A·n_th_) and Auger (~ C·n_th_^3^) current densities.

**Figure 5:**
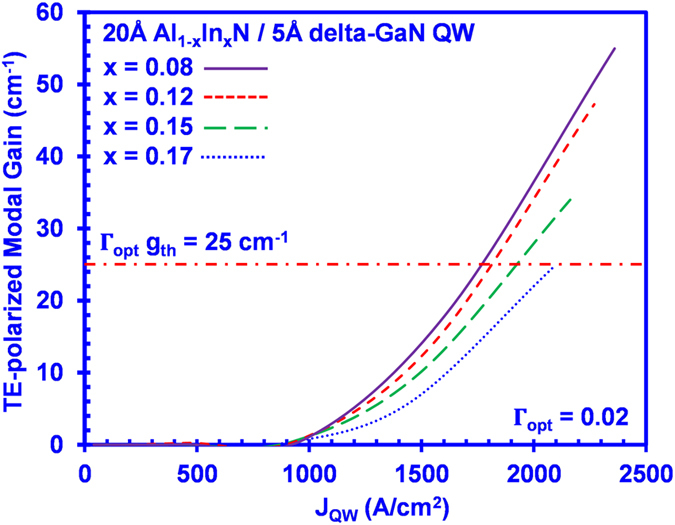
Peak TE-polarized modal gain as a function of current density in the active region (J_QW_ = J_rad_ + J_non-rad_) for 20 Å Al_1–x_In_x_N/5 Å delta-GaN QW.

In order to analyze the impact on devices, our study then takes into account the radiative recombination current density (J_rad_) and non-radiative recombination current density (J_non-rad_) in the QW active region. Note that the total injected current density (J_total_) in the devices will depend on the injection efficiency (*η*_inj_)^38^, which represents the fraction of the injected current density that recombines in the QW [*η*_inj_ = (J_rad_ + J_non-rad_)/J_total_]. Figure 5 shows the peak TE modal gain as a function of total recombination current density in the QW (J_QW_ = J_rad_ + J_non-rad_) for the AlInN-delta-GaN QWs. The reported values for the Auger coefficient C range from ~10^−30^ cm^6^ s^−1^ up to ~ 10^−31^ cm^6^ s^−1^ 28. In our analysis of the J_non-rad_ term, we have employed C = 10^−31^ cm^6^ s^−1^. The monomolecular recombination rate of A = 1 × 10^9^ s^−1^ was used^31^. As shown in fig. 5, to achieve the modal threshold gain of 25 cm^−1^, the threshold current densities (J_QW_th_) of in the AlInN-delta-GaN QW are estimated as ranging from ~ 1750 A/cm^2^ up to ~2100 A/cm^2^, which represent practical lasing threshold current densities in devices.

On the other hand, the total threshold sheet carrier density for the corresponding conventional AlInN QW to overcome the threshold modal gain of 25 cm^−1^ exceeds *n*_*2D_th*_ ~ 1.6 × 10^13^ cm^−2^, which is more than twice of that for the delta-based QW. This significantly higher *n*_*2D_th*_ in the conventional QW active regions results in an order of magnitude higher non-radiative recombination current in the active region (J_non-rad_ ~ A·n_th_ + C·n_th_^3^), which will be impractical for implementation. This work shows the applicability of the AlInN-delta-GaN QW as active region for laser diodes strongly attributed from the reduction in its threshold carrier densities.

**Figure 6:**
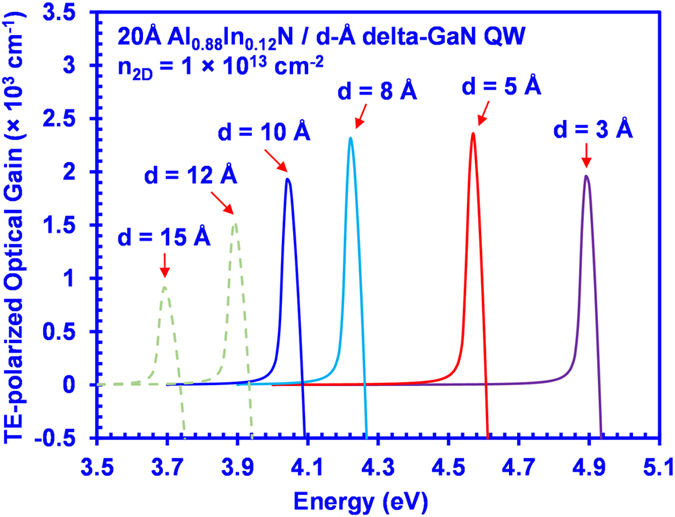
TE peak material gains (g^TE^_peak_) spectra for 20 Å AlInN/d-Å delta-GaN QW with AlN barriers at *n*_*2D*_ = 1 × 10^13^ cm^−2^.

Our analysis has shown that the AlInN-delta-GaN QW yields remarkable advantages with sufficiently high TE-polarized spontaneous emission and optical gain for UV application. However, the TE-polarized spontaneous emission and optical gain spectra of the AlInN-delta-GaN QWs suffer undesirable redshift with increased In-content, as shown in fig. 3(a) and fig. 4(a), which should be carefully treated. Previous studies show the possibility of engineering the delta-GaN layer in the QW structure for achieving various emission wavelengths^23^. In our study, the TE-polarized optical gain spectra were calculated for 20 Å Al_0.88_In_0.12_N/delta-GaN QWs with various delta-GaN layer thickness (*d*). Figure 6 presents the plot of the TE-polarized optical gain spectra of the 20 Å Al_0.88_In_0.12_N/d-Å delta-GaN QW with *d* varying from 3 Å up to 15 Å, at a sheet carrier density of *n*_*2D*_ = 1 × 10^13^ cm^−2^. As seen from fig. 5, the thickness of the delta-GaN layer can be engineered so that the peak TE optical gain can be shifted correspondingly, as well as the emission wavelength. Specifically, reducing the delta-GaN layer thickness to 3 Å shifts the emission wavelength down to ~250 nm with a TE optical gain peak of ~2000 cm^−1^. Although the TE-polarized peak optical gain is reduced as the thickness of delta-GaN layer decreases, it is still much larger than that of the conventional AlInN QW structure. Note that the results for AlInN-delta-GaN QW shown in fig. 6 correspond to a 12% Indium composition, which could be further reduced if lower emission wavelength is desired. Hence, the optimization of the delta-GaN layer thickness is instrumental for achieving the desired emission wavelength along with high TE-polarized optical gain in the AlInN-delta-GaN QW structure.

It is also important to point out that AlInN alloys with various In-content have been grown on GaN template with metalorganic chemical vapor deposition (MOCVD) technique^21^. The growth of AlInN layer can be carried out at temperatures in the range from 750 °C to 860 °C, which is compatible with the growth of the GaN layer^21^. In addition, recent MOCVD work demonstrated the growth of AlN/GaN superlattice structures with thin GaN layer (~0.9–2.5 monolayers)^30^, indicating that the growth of delta-GaN layer can be practically implemented in the AlInN-delta-GaN structure. However, it is also important to note that the growth of AlInN layer is still in the early stage where technical challenges remain to synthesize AlInN alloy with more than 20% In-content^21^. While further optimizations of the growth conditions will still be required to realize the AlInN-delta-GaN structure, the feasibility of the AlInN material growth implies the strong potential of implementing the alloy in the deep UV device applications. The key idea of this work is to illustrate the potential of using the AlInN/delta-GaN QW structure as the active region material, which enables the potential solution to overcome the valence bands crossover issue in the AlN-based devices by applying a relatively low In-composition AlInN alloy.

## Conclusion

In summary, the spontaneous emission and gain characteristics of the AlInN-delta-GaN QWs are analyzed for high performance deep UV light emitters. Our analysis shows that the AlInN-delta-GaN QWs yields ~4 and ~6 times enhancement of the dominant TE-polarized spontaneous emission rate and optical gain, respectively. These significant improvements are attributed to the valance band rearrangement and larger optical transition matrix elements in the AlInN-delta-GaN QWs compared with the conventional AlInN QWs. In addition, our analysis suggests that further optimizations of the delta-GaN layer in the AlInN-delta-GaN are crucial to realize the high performance AlInN-based deep UV emitter devices with the desired emission wavelength. This work shows a potential solution of using the low In-content AlInN-delta-GaN QW structure to achieve sufficiently high dominant TE-polarized spontaneous emission rates and optical gains for the high performance AlN-based UV devices.

## Additional Information

**How to cite this article**: Tan, C.-K. *et al.* Large Optical Gain AlInN-Delta-GaN Quantum Well for Deep Ultraviolet Emitters. *Sci. Rep.* **6**, 22983; doi: 10.1038/srep22983 (2016).
